# An adaptive method for cDNA microarray normalization

**DOI:** 10.1186/1471-2105-6-28

**Published:** 2005-02-11

**Authors:** Yingdong Zhao, Ming-Chung Li, Richard Simon

**Affiliations:** 1 Biometric Research Branch, National Cancer Institute, National Institutes of Health, Rockville, Maryland, USA; 2 The EMMES Corporation, Rockville, Maryland, USA

## Abstract

**Background:**

Normalization is a critical step in analysis of gene expression profiles. For dual-labeled arrays, global normalization assumes that the majority of the genes on the array are non-differentially expressed between the two channels and that the number of over-expressed genes approximately equals the number of under-expressed genes. These assumptions can be inappropriate for custom arrays or arrays in which the reference RNA is very different from the experimental samples.

**Results:**

We propose a mixture model based normalization method that adaptively identifies non-differentially expressed genes and thereby substantially improves normalization for dual-labeled arrays in settings where the assumptions of global normalization are problematic. The new method is evaluated using both simulated and real data.

**Conclusions:**

The new normalization method is effective for general microarray platforms when samples with very different expression profile are co-hybridized and for custom arrays where the majority of genes are likely to be differentially expressed.

## Background

Microarray technology provides simultaneous measurements of expression levels for thousands of genes. Each step from sample preparation to data analysis, however, contains potential sources of bias and variability. Proper normalization adjusts for differences which interfere with the comparison of intensities of different labels at a given probe and with the comparison of intensities of corresponding probes on different arrays. Proper data normalization should allow for the comparison of expression levels across different arrays. Subsequent data analysis results are heavily dependent on effective normalization.

Normalization issues differ for dual-labeled platforms compared to single labeled platforms such as the Affymetrix GeneChip arrays. In this paper we address normalization for dual-labeled arrays with either cDNA or oligonucleotide probes. The objective of normalization for dual-labeled arrays is to correct for differences in intensities for the two labels on the same array. These differences arise from factors such as differences in sample concentrations, differences in photomultiplier tube setting, and differences in the affinity of the two labels for DNA.

Median or mean based global normalization methods use a single normalization factor applied to all genes on the array to adjust for labeling bias [1,2]. Such methods are widely used because of their simplicity. Intensity-based and location-based methods take into account intensity and spatial dependence on dye bias normalization factors [3,4]. Both global and intensity/location based normalization methods assume that most of the genes are not differentially expressed between the two samples hybridized on the array, and that for the differentially expressed genes, the direction of the difference is symmetric between the two samples. In many important cases, however, these assumptions are not appropriate because: 1) more than half of the genes are differentially expressed on the array; 2) the numbers of over- and under-expressed genes on the array are unequal; 3) only genes of specific biological interest are selected to make a customized array, which are highly variable across the samples. In the above cases, the global normalization methods and intensity/location based normalization methods become less accurate and a more sophisticated method is needed [5,6].

There are some methods which attempt to adaptively identify the subset of 'housekeeping' genes [6-8]. These methods require multiple arrays in order to identify the 'housekeeping' gene set, which does not always exist.

Newton *et al. *proposed a Gamma-Gamma-Bernoulli model for identifying differentially expressed genes in dual labeled arrays [9]. We have generalized Newton's model and here propose an adaptive method based on three-component mixture model for normalization of dual labeled microarray data.

## Results

As described in the Methods section, we have applied our adaptive method to both the simulated data and real data. We have also compared our method with the global method and the intensity-based lowess method.

**Figure 1:**
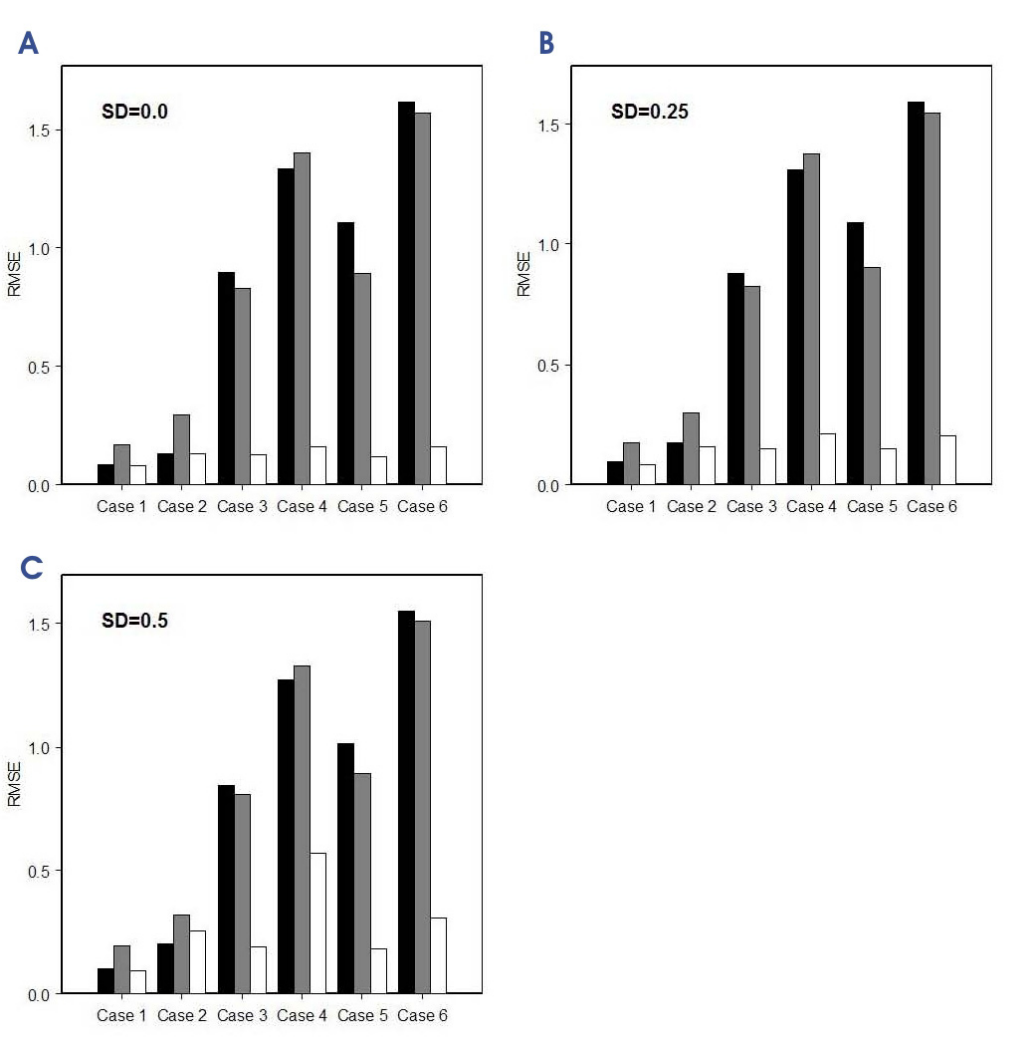
Bar plots show comparison of RMSEs by using the global method (black bar), the lowess method (grey bar), and the adaptive method (white bar) for normalization with simulated data generated from a mixture model with *c *= 1.5, *a *= 118, *a*_0 _= 410, *γ *= 31, *γ*_1 _= 23, and *γ*_2 _= 29 at three different noise levels (A) SD = 0; (B) SD = 0.25; and (C) SD = 0.50.

Results of the simulation studies are shown as bar plots in Figure 1. Figure 1A shows the comparison of our adaptive method, the global method and the lowess method when no noise was added. When the majority of genes in the array were non-differentially expressed (Case 1), or the numbers of over- and under-expressed genes on the array were equal (Case 2), the root mean squared error (RMSE) of the adaptive method was comparable with the other two methods; all were very small. When the array contained unequal numbers of over- and under-expressed genes and when the majority of genes were differentially expressed (Cases 3–6), the RMSEs of the global normalization method and the lowess method were much larger than those of the adaptive method. The differences ranged from around a two fold difference (0.895 in log_2 _scale) when the number of under-, null, and over-expressed genes were 200, 100, and 100, to more than a three fold difference (1.617 in log_2 _scale) when the number of under-, null, and over-expressed genes were 200, 50, and 50. The RMSEs for the adaptive method ranged from 0.078 to 0.159 in log_2 _scale.

**Figure 2:**
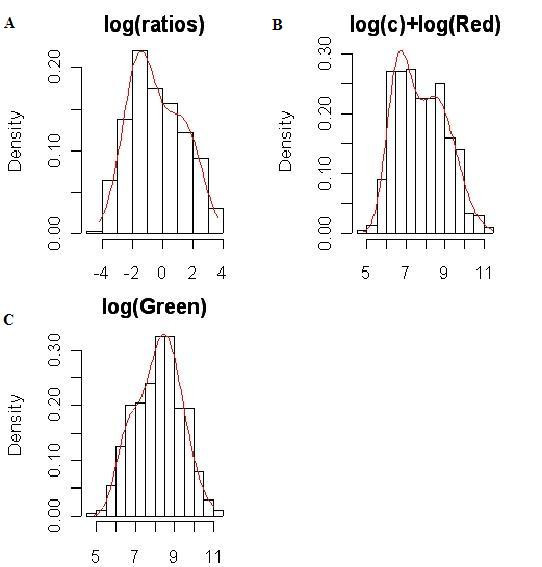
Histograms and the estimated densities of log(ratio) and log(intensity) for a set of simulated data generated from a mixture model with *c *= 1.5, *a *= 118, *a*_0 _= 410, *γ *= 31, *γ*_1 _= 23, and *γ*_2 _= 29. The superimposed curve on each plot is generated from the fitted model.

We compared the histogram of observed intensities to the fitted marginal density from the adaptive method as a simple check to see whether the proposed model and the estimation procedure are in line with available data. Figure 2 shows the histograms of log(ratio) and log intensities of red and green channels of the simulated data, and the curve in each plot is the estimated density obtained from the fitted model. It is seen the data fits to the model quite well.

Gaussian noise with SD of 0.25 and 0.50 were added so that the data was not generated from the same model used for analysis with the adaptive method. The RMSEs of the global normalization method and the lowess method remained large, while the RMSEs of the adaptive method remained small, ranging from 0.083 to 0.569 on the log_2 _scale (Figure 1B and 1C).

In the above simulation, no apparent groups could be seen in the histograms of log(ratio) (Figure 2A). Better results for the adaptive method were also obtained for a simulation case where the three groups (under-expressed, non-differentially expressed, and over-expressed) are apparent in the histogram of log(ratio). The results can be seen in Figure 4 and Figure 5 [see Additional files 3, 4].

**Figure 3:**
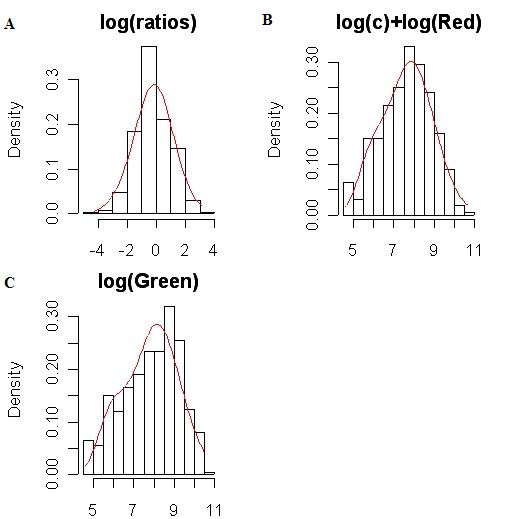
Histograms and the estimated densities of log(ratio) and log(intensity) for a set of real data generated from array svcc109. The superimposed curve on each plot is generated from the fitted model.

Results comparing RMSEs for the adaptive method, the global method and the lowess method with real data are shown in Table 2. The RMSEs of the adaptive method on data generated from ten different arrays ranged from 0.128 to 0.529, in comparison with RMSEs of around 1.0 using the global normalization method. The average RMSE (0.607) of the lowess method is almost two times that of our adaptive method (0.328), although the lowess method performed better than the global method (average RMSE = 1.016). Figure 3 shows the histograms of log(ratio) and log intensities of red and green channels of the real data, and the curve in each plot is the estimated density from the adaptive method.

## Discussion

In this paper, we propose a new method for normalization of dual-labeled arrays in cases where the number of differentially expressed genes is substantial and not necessarily symmetric in direction. The method performed effectively with both simulated and real data.

We started our model building initially by introducing an unknown constant *c *into Newton's Gamma-Gamma-Bernoulli model [9]. The mixture model consisted of two groups: non-differentially expressed genes (Equation 1A) and differentially expressed genes (Equation 1B).

log(*cR*_*k*_) ~ *Gamma*(*a*, *s*_*k*_)

log(*G*_*k*_) ~ *Gamma*(*a*, *s*_*k*_)     (1A)

*s*_*k *_~ *Gamma*(*a*_0_, *γ*)

log(*cR*_*k*_) ~ *Gamma*(*a*, 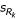)

log(*G*_*k*_) ~ *Gamma*(*a*, 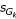)     (1B)


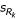
 ~ *Gamma*(*a*_0_, *γ*)


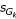
 ~ *Gamma*(*a*_0_, *γ*)

We found that when the differential expression was symmetric between the two samples, the model worked well. However, the error increased significantly when the ratio of the numbers of under- to over-expressed genes shifted from 1.

In order to make the model more flexible, we modified the model by assigning different scale factors *γ*_*R *_and *γ*_*G *_for the red channel and green channel intensities. For this modified two-component mixture, the error still remained large. We then extended the model into a three-component mixture model listed as Equations 8A-8C in the additional material [see Additional file 2]. The model was then quite flexible but there were too many parameters that needed to be optimized. After we tested it with simulation data and real data, we found the estimated model was not stable and difficult to optimize. We finally simplified the model to our final model given by Equations 2A-2C (see Methods section). When applying it to real data or simulated data, the estimates converged well close to globe optima. When different start points were used, the optimizations remained relatively robust.

Evaluation of normalization methods can be difficult since the true normalization factors are unknown with real data for custom arrays. We avoided this problem by synthesizing customized arrays based on real data for standard arrays containing thousands of genes. In order to make the distribution of each component group look smoother, we allowed certain range of overlap between the adjacent groups. Additional sampling method was tried to divide the whole distribution range into many non-overlapping intervals. In each interval the number of genes sampled increased when the absolute value of log_2_(ratio) became larger (Table 3 [see Additional file 6]). The model fitting results using data generated by this sampling method are listed in Table 4 [see Additional file 7] and Figure 6 [see Additional file 5].

We compared our adaptive method with the global method and the intensity-based lowess method. The lowess method assumes that in each intensity interval either the majority of genes are non-differentially expressed or the numbers of up- and down-regulated genes are equal. The global median normalization makes these assumptions only over the array as a whole. It is not surprising that our method performed much better that the above two methods, because the global median method only works well when the assumptions are valid while the intensity-based lowess method is only effective when there are intensity-dependent biases.

Correlation structure is complicated for the thousands of genes on a microarray. In our model, the intensity of each channel is conditionally independent given the scale parameter, but not marginally independent.Therefore, we are not assuming the intensities in two channels are independent. Although we did not generate correlated genes in our simulated data sets, correlations of genes do exist in the real data sets we tested. Spatial correlations are also possible but our method is not designed for that purpose. Yang *et al. *proposed using the lowess normalization separately within each grid on the array [3]. Our algorithm could be similarly applied within each grid to control for spatial effects.

Limited simulations were performed in this study. We also tried to use real data to test our method. Since an appropriate data set with known normalization factor was not available, we synthesized such data sets by sub-setting large arrays in which the true normalization factor could be accurately estimated. In the process of synthesizing such small arrays we had to choose an empirical threshold to stratify the differentially expressed genes and non-differentially expressed genes. Although we do not believe that the superiority shown for our algorithm depends critically on the threshold chosen nor on details of the synthesis, it would be preferable to evaluate the algorithm on real data sets with know normalization factors.

Although our method is designed for dual-labeled cDNA array, it can be extended to single channel Affymetrix chip data. The most popular normalization method for the Affymetrix chip compares each array to a single base line array for probe set summaries. The assumptions behind the normalization method are that the majority of the genes are non-differentially expressed and the numbers of over- and under-expressed genes are roughly equal; the same assumptions as those for dual-labeled cDNA arrays. We could treat the base line array as the 'reference channel' and the other array as the 'test' channel and apply our algorithm to probe set summaries. For Affymetrix chip data, there are multiple base pairs in a probe set and each probe has an intensity measurement. Several alternative normalization methods of Affymetrix arrays utilize the probe level information. For example, method based on an 'invariant set' proposed by Li and Wong assumes that a probe of a non-differentially expressed genes in two arrays to have similar ranks and uses an iterative procedure to identify the invariant set which presumably consists of points from non-differentially expressed genes [10].

## Conclusions

Our new normalization method does not require that the majority of genes be non-differentially expressed, and doesn't require multiple array replicates, dye swaps, spiked controls, or housekeeping genes. It appears much more effective than standard methods when the numbers of over- and under-expressed genes are unequal, and the majority of the genes are differentially expressed. It can be very useful for general microarray platforms when samples with very different expression profile are co-hybridized and for custom arrays where the majority of genes are likely to be differentially expressed. In both of these settings, standard normalization methods are problematic.

## Methods

### Model

We define true intensities for a specific gene *k *in two channels as *R*_*k *_(red) and *G*_*k *_(green). Let *c *be a positive constant which is related to the normalization constant. The observed intensities for gene *k *in two channels are *cR*_*k *_and *G*_*k*_. We assume the logarithm of intensity in each channel has a *Gamma *distribution. The genes on the array belong to three different groups: 1) non-differentially expressed; 2) under-expressed; and 3) over-expressed. The overall data will be fitted into a mixture model listed below.

For a non-differentially expressed gene *k*,

log(*cR*_*k*_) ~ *Gamma*(*a*, *s*_*k*_)

log(*G*_*k*_) ~ *Gamma*(*a*, *s*_*k*_)     (2A)

*s*_*k *_~ *Gamma*(*a*_0_, *γ*).

For an under-expressed gene *k*,

log(*cR*_*k*_) ~ *Gamma*(*a*, 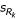)

log(*G*_*k*_) ~ *Gamma*(*a*, 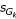)     (2B)


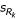
 ~ *Gamma*(*a*_0_, *γ*_1_)


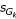
 ~ *Gamma*(*a*_0_, *γ*_2_).

For an over-expressed gene *k*,

log(*cR*_*k*_) ~ *Gamma*(*a*, 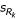)

log(*G*_*k*_) ~ *Gamma*(*a*, 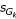)     (2C)


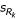
 ~ *Gamma*(*a*_0_, *γ*_2_)


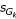
 ~ *Gamma*(*a*_0_, *γ*_1_).

In the above *Gamma *distributions, the parameters *a *and *a*_0 _are shape factors, and the parameters *s*_*k*_, *γ*, *γ*_1_,*γ*_2_, 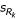, 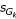 are scale factors. The parameters *a*, *a*_0_, *γ*, *γ*_1_, *γ*_2 _will be estimated from the data.

Let *p*_*u*_(*R*_*k*_, *G*_*k*_), *p*_*o*_(*R*_*k*_, *G*_*k*_) and *p*_*n*_(*R*_*k*_, *G*_*k*_) be the densities of (*R*_*k*_, *G*_*k*_) for under-expressed, over-expressed and non-differentially expressed genes, respectively. The joint distributions of (*R*_*k*_, *G*_*k*_) in three groups can be derived as follows [details see Additional file 1]:


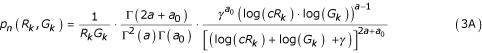



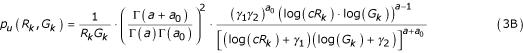



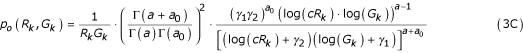


Let *θ *denote the unknown parameter vector (*a*, *a*_0_, *γ*, *γ*_1_, *γ*_2_, *c*), which can be estimated by maximizing the likelihood function of observed data. We used the EM algorithm [11] for this maximization. Let *p*_1 _be the proportion of under-expressed genes and *p*_2 _be the proportion of over-expressed genes. We define indicator binary variable *z*_*k*1 _to be 1 if the *k*th gene is under expressed, 0 otherwise; and *z*_*k*2 _to be 1 if the *k*th gene is over expressed, 0 otherwise. The *complete-data loglikelihood *for all spots can be derived as follows,


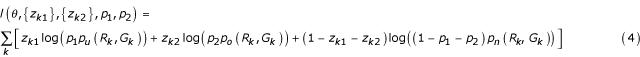


In the M-step, we first take derivative on Equation (4) with respect to *p*_1 _and *p*_2_. This yields


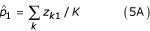



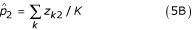


where *K *is the total number of genes on the array.

To maximize Equation (4), we only need to maximize Equation (6) because the left out terms do not depend on the parameter *θ*.


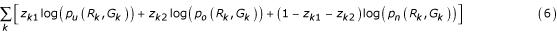


In the E-step, we compute the conditional expectations of *z*_*k*1 _and *z*_*k*2 _given the other parameters from the M-step.


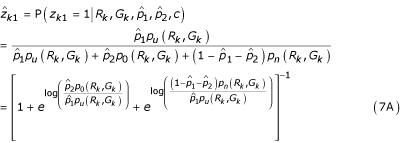



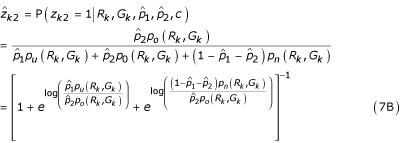


Once the constant *c *was obtained, the normalization constant for log intensity ratio data can be calculated as log(1/*c*).

## Model evaluation

Simulation studies were performed by generating two channel intensities from the mixture model with *c *= 1.5, *a *= 118, *a*_0 _= 410, *γ *= 31, *γ*_1 _= 23, and *γ*_2 _= 29. Six scenarios were included using different proportions of non-differentially expressed genes and different ratios of under- to over- expressed genes, as listed in Table 1.

One hundred data sets were generated for each scenario and the RMSE between the estimated log_2_(1/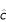) and the true log2(1/*c*) was calculated. The global method takes the median log_2_(ratio) of all genes in each data set as the normalization factor. The lowess method performs robust locally linear fits of M-A plot and corrects the biases that are dependent on spot intensity [3]. The RMSE between the normalized log_2_(ratio) using the lowess method and the normalized log_2_(ratio) using the global method with the true normalization factor (log_2_(1/*c*)) for all genes was also calculated for the same 100 data sets.

Gaussian white noise was also added when generating the simulated data. We used standard deviation of 0.25 in log_2 _scale to reflect the experimental noise in inbred strains of mice or cell line data and 0.5 in log_2 _scale to reflect a larger experimental noise in human tissue data [12].

*In-silico *studies were performed on real data. We tested the method on ten arrays from publicly available breast cancer data [13]. Each array consists of 9216 genes. The common reference sample was a pool of RNA isolated from 11 different cultured cell lines (green channel, labeled with Cy3). RNA from tissues of breast cancer patients were used in the test channel (red channel, labeled with Cy5). The array was first normalized by the global normalization method. The median log_2_(ratio) of all genes was considered as the true normalization factor *c*. The genes were then divided into three groups: over-expressed genes (log_2_(ratio)>1), non-differentially expressed genes(-1.5<log_2_(ratio)<1.5), and under-expressed genes (log_2_(ratio)<-1). We randomly sampled a specified number of genes from each group (100 non-differentially expressed genes, 200 under-expressed and 100 over-expressed genes) and then combined them into an *in-silico *array. We constructed 100 datasets for each of the 10 arrays in this way and the RMSE between the estimated log_2_(1/ 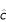) and the true log_2_(1/*c*) was calculated.

## Supplementary Material

Additional File 3 Figure 4: Bar plots show comparison of RMSE by using the adaptive method (black bar) and global method (grey bar) with simulated data generated from a mixture model with *c *= 1.5, *a *= 90, *a*_0 _= 120, *γ *= 8, *γ*_1 _= 6, and *γ*_2 _= 10 at three different noise levels (A) SD = 0; (B) SD = 0.25; and (C) SD = 0.50. Click here for file

Additional File 4 Figure 5: Histograms and the estimated densities of log(ratio) and log(intensity) for a simulated data of a mixture model with *c *= 1.5, *a *= 90, *a*_0 _= 120, *γ *= 8, *γ*_1 _= 6, and *γ*_2 _= 10. The superimposed curve on each plot is generated from the fitted model. Click here for file

Additional File 2 Equations 8A-8C: a three-component mixture model. Click here for file

Additional File 5 Figure 6: Histograms and the estimated densities of log(ratio) and log(intensity) for a set of real data generated from array svcc109. The superimposed curve on each plot is generated from the fitted model. The procedure to generate the data was described in the paper and the sampling rate was shown in Table 3 [see Additional file 6]. Click here for file

Additional File 6 Table 3: Different number of genes sampled in each interval. Click here for file

Additional File 7 Table 4: Comparison of RMSE by using the adaptive method and global method with real data by a different sampling method. The procedure to generate the data was described in the paper and the sampling rate was shown in Table 3 [see Additional file 6]. Click here for file

Additional File 1 Derivation of joint distributions of (R, G) for *p*_*n*_, *p*_*u*_, and *p*_*o *_ Click here for file

## Figures and Tables

**Table 1:** Six scenarios using different proportions of non-differentially expressed genes and different ratios of under- to over-expressed genes with simulated data.

	Number of genes		
Case	Under-expressed	Non-differentially expressed	Over-expressed
1	100	500	100
2	100	100	100
3	200	100	100
4	200	100	50
5	200	50	100
6	200	50	50

**Table 2:** Comparison of RMSEs by using the global method, the lowess method, and the adaptive method for normalization with real data.

Case	Array ID	Global	Lowess	Adaptive
1	svcc134	1.082	0.600	0.315
2	svcc104	1.023	0.601	0.508
3	svcc120	1.005	0.552	0.435
4	svcc64	0.999	0.593	0.516
5	svcc106	0.967	0.704	0.128
6	svcc89	1.018	0.593	0.284
7	svcc109	1.014	0.577	0.264
8	svcc103	1.011	0.653	0.138
9	svcc98	1.022	0.631	0.159
10	svcc82	1.017	0.567	0.529
Average RMSE		1.016	0.607	0.328
