# Supplementary figures and images for: An adaptive method for cDNA microarray normalization
Source: BMC Bioinformatics. 2005 Feb 11;6:28. doi: 10.1186/1471-2105-6-28 (PMC552315; doi:10.1186/1471-2105-6-28)

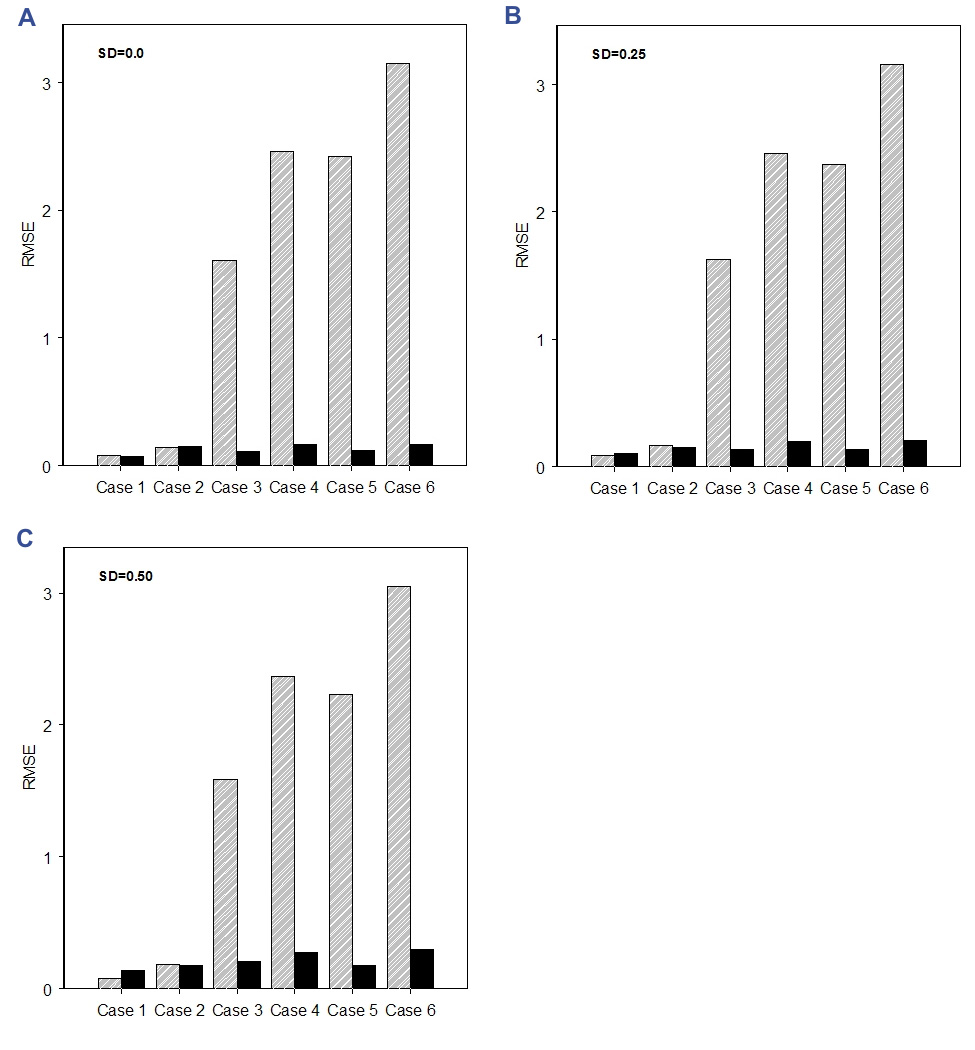

Supplement: Additional File 3 — Figure 4: Bar plots show comparison of RMSE by using the adaptive method (black bar) and global method (grey bar) with simulated data generated from a mixture model with c = 1.5, a = 90, a0 = 120, γ = 8, γ1 = 6, and γ2 = 10 at three different noise levels (A) SD = 0; (B) SD = 0.25; and (C) SD = 0.50. [file 1471-2105-6-28-S3.jpeg]

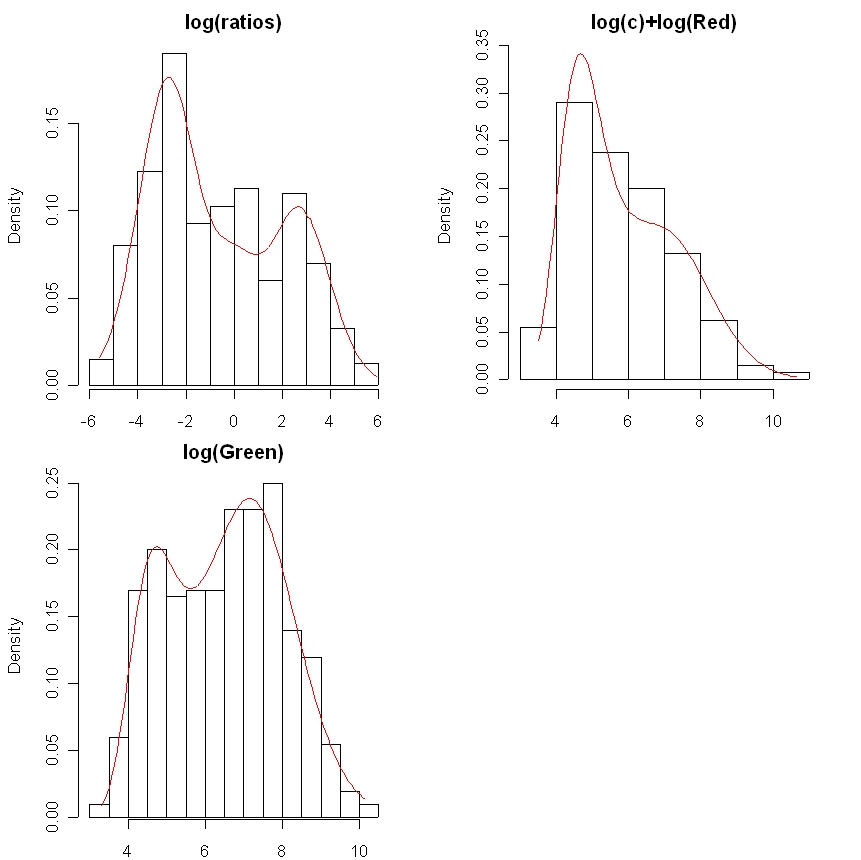

Supplement: Additional File 4 — Figure 5: Histograms and the estimated densities of log(ratio) and log(intensity) for a simulated data of a mixture model with c = 1.5, a = 90, a0 = 120, γ = 8, γ1 = 6, and γ2 = 10. The superimposed curve on each plot is generated from the fitted model. [file 1471-2105-6-28-S4.jpeg]

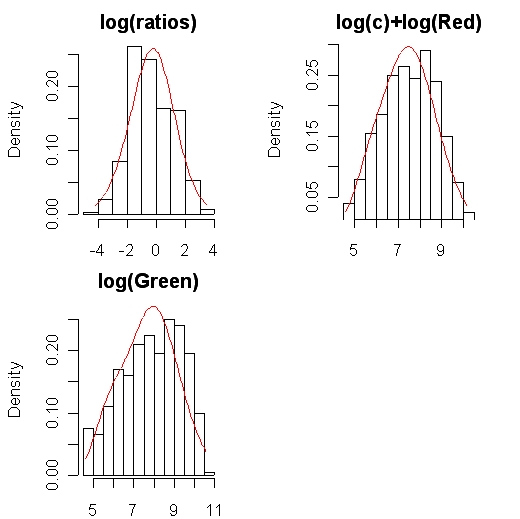

Supplement: Additional File 5 — Figure 6: Histograms and the estimated densities of log(ratio) and log(intensity) for a set of real data generated from array svcc109. The superimposed curve on each plot is generated from the fitted model. The procedure to generate the data was described in the paper and the sampling rate was shown in Table 3 [see Additional file 6]. [file 1471-2105-6-28-S5.jpeg]
