# Supplementary material for: An adaptive method for cDNA microarray normalization
Source: BMC Bioinformatics. 2005 Feb 11;6:28. doi: 10.1186/1471-2105-6-28 (PMC552315; doi:10.1186/1471-2105-6-28)
Supplement: Additional File 2 — Equations 8A-8C: a three-component mixture model. [file 1471-2105-6-28-S2.pdf]

Equations 8A-8C: a three-component mixture model

$$\log(cR_k) \sim Gamma(a, s_k)$$
$$\log(G_k) \sim Gamma(a, s_k) \quad (8A)$$
$$s_k \sim Gamma(a_0, \gamma)$$

$$\log(cR_k) \sim Gamma(a, s_{R_k})$$
$$\log(G_k) \sim Gamma(a, s_{G_k}) \quad (8B)$$
$$s_{R_k} \sim Gamma(a_0, \gamma_{R1})$$
$$s_{G_k} \sim Gamma(a_0, \gamma_{G1})$$

$$\log(cR_k) \sim Gamma(a, s_{R_k})$$
$$\log(G_k) \sim Gamma(a, s_{G_k}) \quad (8C)$$
$$s_{R_k} \sim Gamma(a_0, \gamma_{R2})$$
$$s_{G_k} \sim Gamma(a_0, \gamma_{G2})$$
