# Supplementary material for: An adaptive method for cDNA microarray normalization
Source: BMC Bioinformatics. 2005 Feb 11;6:28. doi: 10.1186/1471-2105-6-28 (PMC552315; doi:10.1186/1471-2105-6-28)
Supplement: Additional File 6 — Table 3: Different number of genes sampled in each interval. [file 1471-2105-6-28-S6.pdf]

Table 3: Different number of genes sampled in each interval.

Range for log_2(ratio)	# of sampled genes
2.50 ---- inf	40
2.00 ---- 2.50	25
1.50 ---- 2.00	20
1.25 ---- 1.50	15
1.00 ---- 1.25	10
0.75 ---- 1.00	10
0.50 ---- 0.75	10
0.25 ---- 0.50	10
0.00 ---- 0.25	10
(-0.25) ---- 0.00	10
(-0.50) ---- (-0.25)	10
(-0.75) ---- (-0.50)	10
(-1.00) ---- (-0.75)	15
(-1.25) ---- (-1.00)	20
(-1.50) ---- (-1.25)	30
(-2.00) ---- (-1.50)	40
(-2.50) ---- (-2.00)	50
-inf ---- (-2.50)	65
Total number of genes	400
