# Supplementary material for: An adaptive method for cDNA microarray normalization
Source: BMC Bioinformatics. 2005 Feb 11;6:28. doi: 10.1186/1471-2105-6-28 (PMC552315; doi:10.1186/1471-2105-6-28)
Supplement: Additional File 7 — Table 4: Comparison of RMSE by using the adaptive method and global method with real data by a different sampling method. The procedure to generate the data was described in the paper and the sampling rate was shown in Table 3 [see Additional file 6]. [file 1471-2105-6-28-S7.pdf]

Table 4: Comparison of RMSE by using the adaptive method and the global method with real data by a different sampling method. The procedure to generate the data was described in the paper and the sampling rate was shown in Table 3 [see Additional file 6].

Case	Array Id	Global	Adaptive
1	svcc134	1.091	0.213
2	svcc104	1.055	0.412
3	svcc120	1.024	0.219
4	svcc64	1.019	0.476
5	svcc106	1.046	0.196
6	svcc89	1.040	0.199
7	svcc109	1.036	0.161
8	svcc103	1.045	0.161
9	svcc98	1.038	0.341
10	svcc82	1.039	0.482
