# Supplementary material for: An adaptive method for cDNA microarray normalization
Source: BMC Bioinformatics. 2005 Feb 11;6:28. doi: 10.1186/1471-2105-6-28 (PMC552315; doi:10.1186/1471-2105-6-28)
Supplement: Additional File 1 — Derivation of joint distributions of (R, G) for pn, pu, and po [file 1471-2105-6-28-S1.pdf]

Derivation of joint distributions of (R, G) for p_n , p_u , and p_o

$$p_n(R,G)=$$
$$=\frac{1}{RG}\int\frac{e^{-\theta\log(cR)}(\log(cR))^{a-1}\theta^a}{\Gamma(a)}\frac{e^{-\theta\log(G)}\log(G)^{a-1}\theta^a}{\Gamma(a)}\frac{e^{-\theta\gamma}\theta^{a_0-1}\gamma^{a_0}}{\Gamma(a_0)}d\theta$$
$$=\frac{1}{RG}\int\frac{e^{-\theta(\log(cR)+\log(G)+\log(\gamma))}\left(\log(cR)\log(G)\right)^{a-1}\theta^{2a+a_0-1}\gamma^{a_0}}{\Gamma^2(a)\Gamma(a_0)}d\theta$$
$$=\frac{1}{RG}\int\frac{e^{-\theta(\log(cR)+\log(G)+\log(\gamma))}\theta^{2a+a_0-1}(\log(cR)+\log(G)+\gamma)^{2a+a_0}}{\Gamma(2a+a_0)}d\theta\bullet\left(\frac{\Gamma(2a+a_0)\left(\log(cR)\log(G)\right)^{a-1}\gamma^{a_0}}{\Gamma^2(a)\Gamma(a_0)(\log(cR)+\log(G)+\gamma)^{2a+a_0}}\right)$$
$$=\frac{1}{RG}\frac{\Gamma(2a+a_0)}{\Gamma^2(a)\Gamma(a_0)}\frac{\gamma^{a_0}(\log(cR)\log(G))^{a-1}}{\left(\log(cR)+\log(G)+\gamma\right)^{2a+a_0}}$$

$$p_u(R,G)=$$
$$=\frac{1}{RG}\int\frac{e^{-\theta\log(cR)}(\log(cR))^{a-1}\theta^a}{\Gamma(a)}\frac{e^{-\theta\gamma_1}\theta^{a_0-1}\gamma_1^{a_0}}{\Gamma(a_0)}d\theta\bullet\int\frac{e^{-\theta\log(G)}(\log(G))^{a-1}\theta^a}{\Gamma(a)}\frac{e^{-\theta\gamma_2}\theta^{a_0-1}\gamma_2^{a_0}}{\Gamma(a_0)}d\theta$$
$$=\frac{1}{RG}\int\frac{e^{-\theta(\log(cR)+\gamma_1)}\theta^{a+a_0-1}(\log(cR)+\gamma_1)^{a+a_0}}{\Gamma(a+a_0)}d\theta\bullet\left(\frac{\Gamma(a+a_0)\left(\log(cR)\right)^{a-1}\gamma_1^{a_0}}{\Gamma(a)\Gamma(a_0)(\log(cR)+\gamma_1)^{a+a_0}}\right)$$
$$\bullet\int\frac{e^{-\theta(\log(G)+\gamma_2)}\theta^{a+a_0-1}(\log(G)+\gamma_2)^{a+a_0}}{\Gamma(a+a_0)}d\theta\bullet\left(\frac{\Gamma(a+a_0)(\log(G))^{a-1}\gamma_2^{a_0}}{\Gamma(a)\Gamma(a_0)(\log(G)+\gamma_2)^{a+a_0}}\right)$$
$$=\frac{1}{RG}\left(\frac{\Gamma(a+a_0)}{\Gamma(a)\Gamma(a_0)}\right)^2\frac{\left(\gamma_1\gamma_2\right)^{a_0}(\log(cR)\log(G))^{a-1}}{\left[\left(\log(cR)+\gamma_1\right)\left(\log(G)+\gamma_2\right)\right]^{a+a_0}}$$

$$p_o(R,G)=$$
$$=\frac{1}{RG}\int\frac{e^{-\theta\log(cR)}(\log(cR))^{a-1}\theta^a}{\Gamma(a)}\frac{e^{-\theta\gamma_2}\theta^{a_0-1}\gamma_2^{a_0}}{\Gamma(a_0)}d\theta\bullet\int\frac{e^{-\theta\log(G)}(\log(G))^{a-1}\theta^a}{\Gamma(a)}\frac{e^{-\theta\gamma_1}\theta^{a_0-1}\gamma_1^{a_0}}{\Gamma(a_0)}d\theta$$
$$=\frac{1}{RG}\int\frac{e^{-\theta(\log(cR)+\gamma_2)}\theta^{a+a_0-1}(\log(cR)+\gamma_2)^{a+a_0}}{\Gamma(a+a_0)}d\theta\bullet\left(\frac{\Gamma(a+a_0)\left(\log(cR)\right)^{a-1}\gamma_2^{a_0}}{\Gamma(a)\Gamma(a_0)(\log(cR)+\gamma_2)^{a+a_0}}\right)$$
$$\bullet\int\frac{e^{-\theta(\log(G)+\gamma_1)}\theta^{a+a_0-1}(\log(G)+\gamma_1)^{a+a_0}}{\Gamma(a+a_0)}d\theta\bullet\left(\frac{\Gamma(a+a_0)(\log(G))^{a-1}\gamma_1^{a_0}}{\Gamma(a)\Gamma(a_0)(\log(G)+\gamma_1)^{a+a_0}}\right)$$
$$=\frac{1}{RG}\left(\frac{\Gamma(a+a_0)}{\Gamma(a)\Gamma(a_0)}\right)^2\frac{\left(\gamma_1\gamma_2\right)^{a_0}(\log(cR)\log(G))^{a-1}}{\left[\left(\log(cR)+\gamma_2\right)\left(\log(G)+\gamma_1\right)\right]^{a+a_0}}$$
